# Whole Genome Sequencing in the Evaluation of Fetal Structural Anomalies: A Parallel Test with Chromosomal Microarray Plus Whole Exome Sequencing

**DOI:** 10.3390/genes12030376

**Published:** 2021-03-06

**Authors:** Jia Zhou, Ziying Yang, Jun Sun, Lipei Liu, Xinyao Zhou, Fengxia Liu, Ya Xing, Shuge Cui, Shiyi Xiong, Xiaoyu Liu, Yingjun Yang, Xiuxiu Wei, Gang Zou, Zhonghua Wang, Xing Wei, Yaoshen Wang, Yun Zhang, Saiying Yan, Fengyu Wu, Fanwei Zeng, Jian Wang, Tao Duan, Zhiyu Peng, Luming Sun

**Affiliations:** 1 Shanghai First Maternity and Infant Hospital, Tongji University School of Medicine, Shanghai 201204, China; jia.zhou71@hotmail.com (J.Z.); xinyaozh@126.com (X.Z.); fighting_vivian@163.com (Y.X.); xiongshiyi@51mch.com (S.X.); yangyingjun@51mch.com (Y.Y.); zougang@51mch.com (G.Z.); weike063053@163.com (X.W.); zhangyun@51mch.com (Y.Z.); wufengyu@51mch.com (F.W.); tduan@yahoo.com (T.D.); 2 BGI Genomics, BGI-Shenzhen, Shenzhen 518083, China; yangziying@bgi.com (Z.Y.); sunjun@bgi.com (J.S.); liulipei@bgi.com (L.L.); liufengxia@bgi.com (F.L.); cuishuge@bgi.com (S.C.); liuxiaoyu@bgi.com (X.L.); weixiuxiu@bgi.com (X.W.); wangzhonghua@bgi.com (Z.W.); wangyaoshen@bgi.com (Y.W.); yansaiying@bgi.com (S.Y.); zengfanwei@bgi.com (F.Z.); 3 Tianjin Medical Laboratory, BGI-Tianjin, BGI-Shenzhen, Tianjin 300308, China; 4 Department of Biology, Faculty of Science, University of Copenhagen, DK-2200 Copenhagen, Denmark; 5 Department of Medical Genetics and Molecular Diagnostic Laboratory, Shanghai Children’s Medical Center, Shanghai Jiaotong University School of Medicine, Shanghai 200127, China; labwangjian@shsmu.edu.cn

**Keywords:** whole genome sequencing, chromosomal microarray, whole exome sequencing, fetal structural anomalies, prenatal diagnosis

## Abstract

Whole genome sequencing (WGS) is a powerful tool for postnatal genetic diagnosis, but relevant clinical studies in the field of prenatal diagnosis are limited. The present study aimed to prospectively evaluate the utility of WGS compared with chromosomal microarray (CMA) and whole exome sequencing (WES) in the prenatal diagnosis of fetal structural anomalies. We performed trio WGS (≈40-fold) in parallel with CMA in 111 fetuses with structural or growth anomalies, and sequentially performed WES when CMA was negative (CMA plus WES). In comparison, WGS not only detected all pathogenic genetic variants in 22 diagnosed cases identified by CMA plus WES, yielding a diagnostic rate of 19.8% (22/110), but also provided additional and clinically significant information, including a case of balanced translocations and a case of intrauterine infection, which might not be detectable by CMA or WES. WGS also required less DNA (100 ng) as input and could provide a rapid turnaround time (TAT, 18 ± 6 days) compared with that (31 ± 8 days) of the CMA plus WES. Our results showed that WGS provided more comprehensive and precise genetic information with a rapid TAT and less DNA required than CMA plus WES, which enables it as an alternative prenatal diagnosis test for fetal structural anomalies.

## 1. Introduction

Congenital fetal anomalies occur in approximately 3% of pregnancies [1], and many of these anomalies have an underlying genetic etiology. Identification of the genetic basis of the anomalies enables informed decision-making, improves perinatal care, and helps to assess recurrence risk for future pregnancies. Chromosomal microarray (CMA) has been broadly adopted to detect copy number variants (CNVs) in prenatal diagnoses with an additional 6% of diagnostic yield over standard karyotyping in fetuses with structural anomalies observed by ultrasound [2,3]. CMA can detect CNVs as small as 10–100 kb in length, depending on the probe density. However, smaller variants, such as single-nucleotide variants (SNVs) and small insertions or deletions (INDELs), which also contribute to a substantial portion of genetic disorders, remain undetectable by this approach [4,5,6,7]. Whole exome sequencing (WES), which detects SNVs, INDELs, and CNVs covering multiple exons, has been proven to be a powerful tool in prenatal diagnosis. In clinical practice, WES can be conducted in CMA-negative cases to further search for single-base lesions. Emerging studies have shown that WES has a detection rate of 8.5% to 10% in fetal structural abnormalities with normal karyotype and CMA results [8,9].

CMA followed by WES considerably increases the diagnostic yield [10,11], and is increasingly accepted as a routine test strategy in clinical practice; however, given the time-sensitive nature of the prenatal stage and the potential inaccessibility of adequate fetal samples, sequential testing is time-consuming and requires a large amount of DNA as input. More importantly, it is unable to detect certain types of variation, such as balanced translocation or noncoding SNVs/INDELs. Whole genome sequencing (WGS) has the potential to detect almost all types of genomic variants with a low input-DNA requirement (≈100 ng) and is proposed to be beneficial in prenatal diagnosis [12,13]. However, except for small case series with a limited number of highly selected fetuses, few studies have tried to evaluate the feasibility and value of WGS in prenatal diagnosis. Talkowski et al. [14] identified precise translocation breakpoints that directly disrupted *CHD7* and *LMBRD1* by using ≈12-fold WGS in an undiagnosed prenatal sample, and this finding could not have been reliably inferred from conventional karyotyping. Choy et al. [15] demonstrated that 30-fold WGS could provide a twofold increase in diagnostic yield (32.0%, 16/50) in fetuses with increased nuchal translucency (≥3.5 mm) compared with routine CMA and/or karyotyping (16.0%, 8/50). The additional diagnoses by WGS include seven (14%, 7/50) cases with pathogenic or likely pathogenic (P/LP) SNVs/INDELs and two cryptic insertions and two inversions.

To evaluate the utility of WGS in prenatal diagnosis for fetal structural anomalies, we prospectively applied trio WGS and CMA in parallel in 111 fetuses with structural or growth anomalies, and performed WES when CMA was negative (CMA plus WES). Then, we compared the results of WGS with CMA plus WES.

## 2. Materials and Methods

### 2.1. Study Design and Procedure 

**Figure 1:**
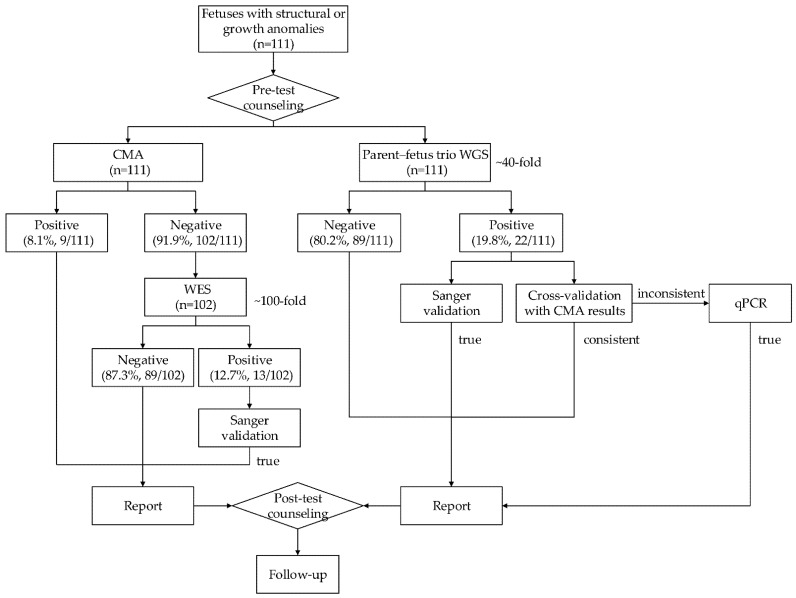
Flowchart of the study. A total of 111 fetuses with structural or growth anomalies were subjected to two test strategies: trio genome sequencing (whole genome sequencing (WGS)) and chromosomal microarray (CMA) plus exome sequencing (whole exome sequencing (WES)). WES was sequentially performed in only 102 fetuses with negative CMA results. The positive/negative rates are provided in each box.

The flowchart of this study is presented in Figure 1. The parents of 111 fetuses with structural or growth anomalies identified by sonographic examination and indicated for prenatal genetic tests were recruited from Shanghai First Maternity and Infant Hospital. Fetal samples were obtained through an invasive diagnostic procedure such as amniocentesis, chorionic villus sampling, or cordocentesis. Parental peripheral blood samples were collected. Samples from fetuses were subjected to two test strategies: parent–fetus trio WGS and CMA plus WES. WES was sequentially performed only in samples with negative CMA results. P/LP SNVs/INDELs were validated by Sanger sequencing, while P/LP CNVs identified by WGS were cross-validated with the results of CMA. Quantitative polymerase chain reaction (qPCR) was conducted for additional P/LP CNVs identified by WGS. Genetic counseling was performed both before and after the prenatal genetic tests, and pregnant women were informed about the results of the tests they had taken. Pregnancy outcomes were followed up.

### 2.2. WGS

Parental and fetal samples were sequenced concurrently (fetus–parent trios or dyads testing). First, 80–200 ng of genomic DNA from each sample was sheared by the Covaris S220 Focused Ultrasonicator (Covaris, Woburn, MA, USA). The fragmented DNA was further processed with AMPure XP beads (Life Sciences, Indianapolis, IN, USA) to obtain 100–300 bp fragments. Library construction, including end repair, A-tailing, adapter ligation, and 7 cycles of PCR amplification, was subsequently conducted. The PCR products were then heat-denatured to form single-strand DNAs, followed by circularization with DNA ligase, and the remaining linear molecule was digested with the exonuclease. After construction of the DNA nanoballs, paired-end sequencing with 100 bp at each end was carried out for each sample with a minimal read depth of 40-fold on the MGISEQ-2000 platform (MGI, Wuhan, China) [16].

Sequencing reads were aligned to the NCBI37/hg19 assembly using the Burrows–Wheeler Aligner (BWA, version 0.7.17, http://bio-bwa.sourceforge.net/ accessed on 6 March 2021) with default parameters. SNVs and INDELs were called using the Genome Analysis Toolkit (GATK, version 4.0.11, https://github.com/broadinstitute/gatk/releases accessed on 6 March 2021) and annotated by the in-house pipeline BGICG_Anno (version 0.3.9). CNVs and structural variants (SVs) were analyzed using CNVnator (version 0.3.2, https://github.com/abyzovlab/CNVnator accessed on 6 March 2021) and LUMPY (version 0.3.0, https://github.com/arq5x/lumpy-sv accessed on 6 March 2021), respectively. The outputs of these callers were merged into a single-variant call format (VCF) file per trio and annotated with public databases and our in-house databases. The results of the SNV/INDEL, CNV, and SV analyses were integrated and reviewed for the interpretation of pathogenicity.

### 2.3. CMA

CytoScan 750K (Affymetrix, Santa Clara, CA, USA) was the CMA platform used in the prenatal genetic diagnosis center of Shanghai First Maternity and Infant Hospital. A total of 250 ng of DNA was required for routine prenatal CMA testing according to the manufacturer’s protocols [17]. CNVs were analyzed via CHAS 2.0 software (NCBI37/hg19), and the reporting size threshold of the CNVs was set at 100 kb with ≥50 contiguous probes.

### 2.4. WES

WES was also performed by inputting 150–300 ng of genomic DNA from each sample, which was prepared with the same library construction procedure as was used for WGS. Exome capture using the MGIEasy Exome Capture V4 Probe (MGI) was followed by paired-end read sequencing (2 × 100 bp read length) on the MGISEQ-2000 platform with an average depth of ≥100-fold. Exome sequencing data analysis was performed as previously described [18].

### 2.5. Data Interpretation and Reporting

CNVs detected by CMA and WGS were interpreted following standards of the American College of Medical Genetics and Genomics (ACMG) and the Clinical Genome Resource (ClinGen) [19]. Among the large numbers of SNVs/INDELs, we prioritized candidate causative SNVs/INDELs per the following criteria: (1) absent or with a minor allele frequency ≤1% in the databases of Exome Aggregation Consortium (ExAC) and Genome Aggregation Database (gnomAD), corresponding to the variant evidence as PM2; (2) family segregation information that was consistent with the inheritance of the variants (PS2/PM6/PM3); (3) supporting evidence from published literature (e.g., PS1/PS3/PS4/PM5); (4) null variants (PVS1); (5) conservation and predicted impact on coding and noncoding sequence (PP3); and (6) relevance to the fetal clinical phenotype (PP4). All the selected variants were assessed for pathogenicity on the basis of the adapted ACMG guidelines and ClinGen sequence variant interpretation working group per updated recommendations for the ACMG criteria [20,21,22].

All of the candidate causative variants were reviewed by a multidisciplinary team of fetal medicine specialists, genetic counselors, and geneticists. The result that was unanimously agreed upon to explain the fetal phenotype included P/LP variants consistent with the inheritance pattern and phenotype of related disorders, as well as variants of unknown significance (VUS) among Online Mendelian Inheritance in Man (OMIM) disease-causing genes that matched the fetal phenotype and were found in trans with a P/LP variant in an autosomal recessive condition, which was designated positive or diagnostic; otherwise, results were designated negative or undiagnostic. The pregnant women and their partners were informed of the ACMG secondary findings [23], and incidental findings were reported only when they consented in the pretest informed consent process according to the ACMG document [24].

### 2.6. Data Validation

SNVs/INDELs/SVs were validated by Sanger sequencing. For CNV validation, we conducted qPCR for additional P/LP CNVs identified by WGS. The HCMV Real-Time PCR Kit (Liferiver, Shanghai, China) was used for validation of one case that was identified by WGS as suspicious for cytomegalovirus (CMV) infection.

## 3. Results

### 3.1. Study Participants

Between November 2019 and January 2020, 111 fetuses with structural or growth anomalies and 209 matched parental samples were eligible for inclusion in our study. Samples from a total of 320 individuals (106 fetus–parental trios, including 4 sets of twins and 5 fetus–parent dyads) were analyzed by WGS, while the 111 fetal samples were also analyzed by CMA in parallel. Samples from 102 fetuses with negative CMA results were subsequently analyzed by WES. The fetuses were assessed at a median gestational age of 24 (range 16–34) weeks. The results of the Down syndrome maternal serum screening test or noninvasive prenatal screening test were recorded when available. Detailed clinical information is available in Table S1.

### 3.2. Comparison of Clinically Relevant Information Provided by WGS and CMA Plus WES

**Figure 2:**
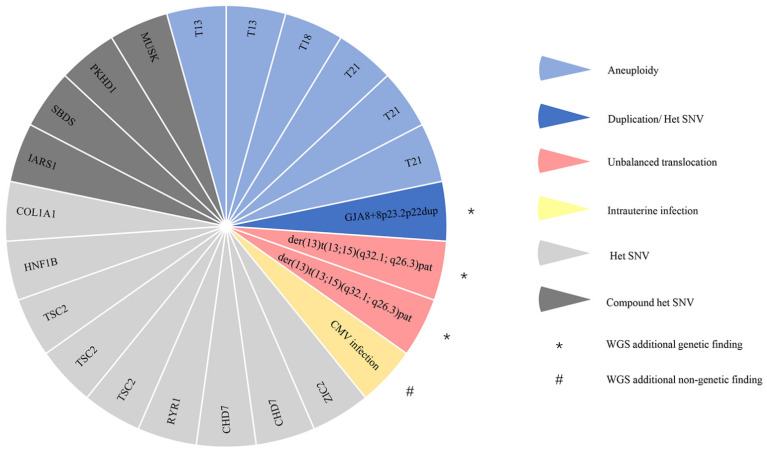
Architecture of a mixed cohort referred for prenatal diagnosis. Each slice of the pie chart represents one individual in the prospective cases analyzed by genome sequencing (WGS) and chromosomal microarray (CMA) plus exome sequencing (WES) where clinically relevant findings were identified. Types of variant are indicated by colors (aneuploidy, light blue; duplication and heterozygous (het) single nucleotide variant (SNV), blue; unbalanced translocation, red; intrauterine infection, yellow; compound heterozygous SNV, light gray; heterozygous SNV, dark gray). Additional genetic findings by WGS are indicated by * and additional nongenetic findings by WGS are indicated by #.

With the CMA plus WES strategy, six (5.4%, 6/111) fetus with aneuploidies (3 with trisomy 21, 2 with trisomy 13, and 1 with trisomy 18) and 3 (2.7%, 3/111) fetuses with P/LP CNVs were identified by CMA (Table 1), while 13 (12.7%, 13/102) fetuses with P/LP SNVs/INDELs were identified by WES (Table 2), providing a diagnostic yield of 19.8% (22/111). In comparison, WGS not only detected all genetic variants in 22 cases diagnosed by CMA plus WES, but also reported 1 additional chromosomal SV, 1 dual-diagnosis case (pathogenic CNVs and SNVs), and 1 case with non-genetic finding (Figure 2).

**Figure 3:**
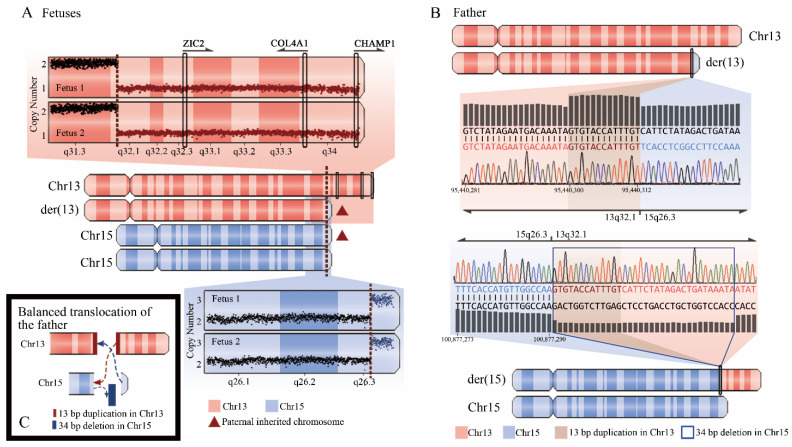
Whole genome sequencing (WGS) identified pathogenic copy number variants (CNVs) in twin fetuses arising from their father’s balanced translocation. (**A**) Distributions of copy number across chromosome 13 and chromosome 15 are shown at the top and bottom, respectively. Dots in red indicate a heterozygous deletion of 19.7 Mb on chromosome 13, and dots in blue indicate a duplication of 1.6 Mb on chromosome 15 in both case 102-1 and case 102-2. The deletion affected three genes that are associated with autosomal dominant developmental disorder. (**B**) The father’s karyotype of chromosome 13 and chromosome 15 are shown at the top and bottom. The distribution of base pair sequencing depth and Sanger sequencing results across the breakpoints on chromosome 13 and chromosome 15 are displayed, respectively, in the middle. There is a templated 13-bp duplication and a 34-bp deletion in the breakpoint junction on chromosomes 13 and 15. (**C**) A schematic drawing of the formation of balanced translocations between chromosomes 13 and 15 in the father.

Among those 22 diagnosed cases, a balanced translocation was directly identified through WGS in the father, and precise translocation breakpoints were also found by checking Integrative Genomics Viewer (IGV) and validated by Sanger sequencing (Figure 3B). There was a templated 13-bp duplication and a 34-bp deletion in the breakpoint junction on chromosomes 13 and 15, respectively (Figure 3C), indicating that the breakpoint repair mechanism might be caused by nonhomologous end joining with occasional deletion during DNA processing before ligation. The father was normal since one breakpoint located in *ADAMTS17* gene associated with autosomal recessive Weill–Marchesani 4 syndrome and the other breakpoint located in a non-disease-causing gene. This couple’s twin fetuses (cases 102-1 and 102-2) were reported with severe hydroderma and pleural effusion, and both CMA and WGS identified a 19.7-Mb deletion on the terminal q-arm of chromosome 13 (13q32.1q34), in which three genes were associated with autosomal-dominant developmental disorder, and a 1.6-Mb duplication on the terminal q-arm of chromosome 15 (15q26.3) (Figure 3A). The CNV findings were consistent with the parental translocations between chromosomes 13 and 15 identified by WGS. The precise genetic diagnosis via WGS allowed for an accurate recurrence risk assessment and effective clinical management of future pregnancies in this family. In addition, the potential for WGS to provide comprehensive clinically relevant information was also reflected in the concurrent detection of CNVs and SNVs. In case 51 with ventricular septal defect, choledochal cyst, ventriculomegaly, and echogenic crystalline lens (suspected congenital cataract), two de novo pathogenic duplications and one de novo pathogenic deletion were detected by both CMA and WGS, however, no parental balanced translocation was identified by carefully analyzing WGS data. In this context, an ≈11.7-Mb duplication on chromosome 8p22-8p23.2 associated with 8p23.1 duplication syndrome [25], whose common features in the reported prenatal cases included congenital heart disease and whose novel features in the reported postnatal cases included ocular anomalies, was considered to explain the fetal phenotype. Thus, WES was not carried out in this case due to the positive CMA finding. However, a clinically relevant de novo pathogenic SNV NM_005267.4, c.593G>A (p. Arg198Gln) in the *GJA8* gene, which was associated with Cataract 1, multiple types (OMIM: #116200), was identified by WGS (Figure 2). This SNV has been reported in three children and their affected mother. All of them were diagnosed with bilateral cataracts soon after birth, and the variant detected in their mother via segregation analysis was de novo [26]. The SNV has also been reported in three affected members of an Indian family with a cataract history and was absent in 400 normal controls [27]. After comprehensive evaluation by clinicians and geneticists, both the SNV and the pathogenic CNV were considered to contribute to the fetus’s phenotype. However, the SNV was missed under the sequential CMA plus WES tests.

**Figure 4:**
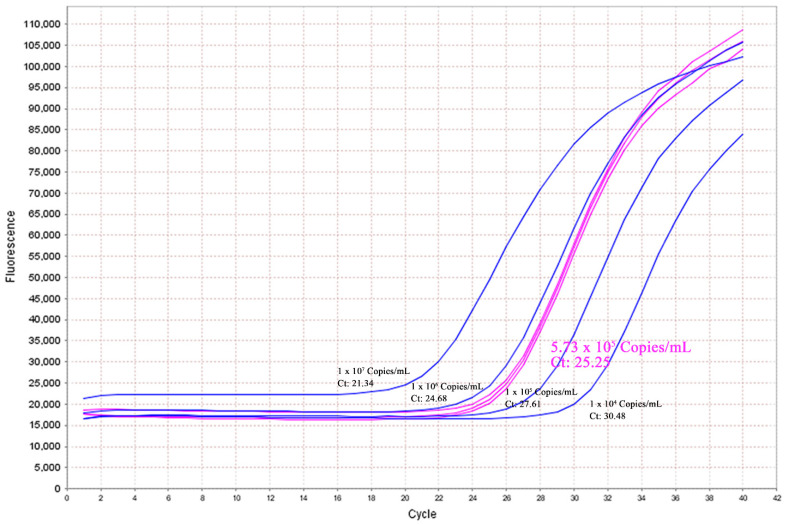
RT-PCR validation of the viral DNA in the amniotic fluid. The load of cytomegalovirus (CMV) was 5.73 × 10^5^ copies/mL, which indicated intrauterine CMV infection in case 5.

In fact, WGS can also provide nonhuman genome information, such as potential pathogens, which we do not usually pay attention to. In our case 5, a fetus with fetal growth restriction was incidentally found to be infected with CMV (the leading infectious cause of newborn malformation [28]) when we noticed that there were some insertions of several dozen base pairs by interpreting the SNVs/INDELs, which was rare in other samples. These reads were only partially mapped to the human genome, and thus we blasted it, which showed 100% similarity to the CMV genome. This case was further validated by amplification of the viral DNA in the amniotic fluid through RT-PCR (Figure 4). Inspired by this case, we developed a bioinformatic pipeline for the detection of pathogens associated with adverse perinatal outcomes, such as CMV, toxoplasma (TOX), and herpes simplex virus 1/2 (HSV 1/2), and analyzed the remaining fetal sample, but no additional positive cases were identified. Although infection was also detected in the WES data via the pipeline developed by us, it may not have detected all pathogens without adding relevant probes due to the capturing nature of WES.

### 3.3. Comparison of the CNV/SNV/INDEL Detection by WGS and CMA Plus WES

Except for some types of variations that are undetectable by CMA and WES, such as balanced translocation and noncoding SNVs/INDELs, we focused on the capacity of WGS to detect CNVs and SNVs/INDELs. We first compared all the CNVs with sizes ≥100 kb detected between CMA and WGS since the reporting threshold of CytoScan 750 K we adopted for CMA was 100 kb. WGS not only detected all the CNVs with sizes ≥100 kb, which were detected by CMA, but also detected additional CNVs that CMA did not (Table S2). In addition, CNVs with sizes smaller than 100 kb were also detected by WGS (Table S3), but none of these small CNVs were determined to be pathogenic. Our data indicated that WGS had a comparable capacity in the detection of CNVs with sizes ≥100 kb and had more potential to detect smaller CNVs than CMA. Second, we compared WES and WGS in terms of their abilities to detect SNVs/INDELs in the coding region. Although the median sequencing depth was higher in WES (144.8-fold ± 28.9 (median ± standard deviation, *n* = 295) vs. 58.0-fold ± 4.2 (*n* = 320)), the coverage at 20-fold of WGS was higher (98.2% ± 1.4% vs. 96.9% ± 1.3%), and WGS detected more candidate variants in the coding region than WES (95% CI, *p* < 0.0001, paired *t*-test, Table S4), which indicated that WGS is more powerful than WES for detecting variants in the coding region.

### 3.4. Subgroup Analysis

We further performed subgroup analyses of the diagnostic yields by WGS and CMA plus WES, and the 111 fetuses that were eligible for WGS were categorized into 10 phenotypic groups on the basis of the types of anomalies detected by ultrasound. The molecular diagnostic rate of WGS among 10 different phenotypic groups ranged from 0% to 39.1% (9/23), as shown in Table S5. The highest diagnostic rate of 39.1% (9/23) was achieved for fetuses with multisystem anomalies, followed by 30.8% (4/13) for fetuses with cardiac anomalies. In these two groups, the diagnosed cases were also both identified by CMA and WES, and thus it is difficult to prejudge the optimal genetic test by phenotype; therefore, in this condition, a comprehensive test may be beneficial.

### 3.5. Impact on Pregnancy Outcome

The effect of genetic diagnosis on pregnancy outcome is shown in Table 3. Follow-up results were available for all 111 fetuses. Among those of 22 diagnosed fetuses, the parents of 21 (95.5%, 21/22) fetuses opted for termination, and the parents of the remaining fetus (4.5%, 1/22) with clubhands, facial abnormalities, omphalocele, and umbilical cord cysts opted to continue the pregnancy. Among those of 89 undiagnosed fetuses, the parents of 34 (38.2%, 34/89) fetuses opted for termination, and the parents of 55 (61.8%, 55/89) fetuses opted to continue the pregnancies (Table S1). We noticed that there were no significant differences in the severities of abnormal fetal phenotypes between the diagnosed group and the undiagnosed group. Thus, in the context of an abnormal fetal phenotype, parents opted for termination of pregnancy significantly more often when they received a positive genetic diagnosis (*p* < 0.0001, Fisher’s exact test), which indicated that the specific genetic diagnosis influenced parental decision-making. Therefore, under the precondition of reporting incidental findings in a limited range, WGS had the advantage of detecting more genetic causes, which may be beneficial to informed decision-making.

### 3.6. Turnaround Time and DNA Requirement

In this study, we tried to shorten the turnaround time (TAT) of each WGS procedure at the expense of cost, so that pregnant women could obtain results faster, while CMA and WES were performed following routine clinical procedures. The TAT for WGS was 18 ± 6 (median ± standard deviation, *n* = 111) days as compared with TAT of 10 ± 2 days (*n* = 111) for CMA and 21 ± 6 days (*n* = 102) for WES. In this regard, WGS could provide a more rapid TAT than CMA plus WES. In addition, a total amount of ≈400 ng (250 ng for CMA and 150 ng for WES) DNA would be required from limited prenatal samples under conventional tests. However, the WGS approach required less fetal DNA (100 ng), which facilitated WGS in prenatal diagnosis, particularly for amniotic fluid samples from early gestational weeks.

## 4. Discussion

Previous studies [8,11,29,30] have demonstrated the clinical utility of CMA and WES in prenatal diagnosis, and a combination of these two approaches for each case has been warranted. In this prospective study, we applied WGS in parallel with CMA plus WES to 111 fetuses with a broad range of structural or growth anomalies. To our knowledge, this is the largest prospective study on the use of high-coverage trio WGS for the prenatal diagnosis of fetal structural abnormalities. As a single test with a rapid TAT (18 ± 6 days) and less DNA required (100 ng), WGS revealed positive genetic findings in 22 fetuses, corresponding to a diagnostic rate of 19.8% (22 in 111). Moreover, WGS provided more comprehensive clinically relevant information than CMA plus WES, including twin fetuses with an unbalanced translocation arising from paternal balanced translocation and one fetus with dual-diagnosis (pathogenic CNVs and SNVs), as well as the intrauterine CMV infection in a growth-restricted fetus. The additional information provided by WGS allowed precise determination of the risk of recurrence and could guide clinical management. However, the cost and data analysis challenges for applying WGS in prenatal diagnosis should not be ignored, and six fetuses with trisomy 21/18/13 who did not undergo serum screening or noninvasive prenatal screening (NIPS) were identified by WGS in our study, reminding us to perform a reliable, rapid, and cost-effective test, such as quantitative fluorescence polymerase chain reaction (QF-PCR) [31], to exclude common fetal chromosomal aneuploidies before performing WGS, especially for fetuses that did not undergo serum screening or NIPS, which seemed to be a more reasonable test path in clinical practice.

WGS not only robustly detects SNVs but also performs well for the detection of CNVs and has the potential to detect SVs [15,32], specific repeat expansions [33,34], and noncoding variants [35,36]. There are possible reasons why the diagnostic rate (19.8%) in this study was lower than that (22% or 32%) in fetuses with structural anomalies in previous reports [11,15], specifically, the prenatal detection rate for P/LP CNVs (2.7%) in our study was significantly lower than those in previous large-scale studies (4.3%–8.2%) [2,11,37]. Apart from the small cohort, one possible reason was that the majority of fetuses (80.2%, 89 of 111 fetuses) in our study received serum screening or noninvasive prenatal screening (NIPS), and common P/LP CNVs were excluded before referral to our fetal medicine unit and prenatal diagnostic center. Another reason was that the classification of genetic variants such as P/LP was more stringent in this study according to the recently published ACMG and ClinGen guidelines [19]. In addition, no specific repeat expansions or noncoding variants were identified by WGS due to the small sample size, which had no contribution to the diagnostic rate, although they only accounted for a small percentage of genetic causes. Of note, the incremental diagnostic yield (12.7%) in fetuses with structural anomalies with CMA-negative results in this study fell within the ranges from 8.5% to 32.0% in case series that included 100 or more fetuses in a previous study [7,8,9,11].

The advantages of applying WGS in prenatal diagnosis were highlighted all the time; except for its ability to cover more types of variation to provide more comprehensive information for the identification of genetic causes, WGS could avoid the dilemma of preselection according to the limited indications in utero, and can naturally present nonhuman genetic information, such as genomes of potential pathogens, in order to indicate infection in the prenatal period, which we do not regularly pay attention to. Notably, if we want to obtain this kind of information, we need to introduce additional methods into the conventional tests or add probes in CMA or WES. However, many obstacles currently hamper the wide utility of WGS in the clinic. The main obstacle is the bioinformatic and interpretation challenges, involving great demand for computational resources, and the needs for analysis and interpretation of the variants by professional multidisciplinary teams consist of fetal medicine specialists, genetic counselors, and geneticists. Ambiguous interpretations will inevitably cause panic and anxiety, however, more incidental findings and huge numbers of VUS variants in the noncoding region can be identified by WGS as opposed to by WES, making it more confusing. Whether they should be reported prenatally is a major concern, and the decision is a particularly challenging one because of the uncertainty associated with these findings, which further increases the complexity of genetic counseling compared to WES and increases difficulty in parental decision-making. Additionally, the choice to report the finding should also be weighed against the risk of missing a potential molecular diagnosis if not reported. The other obstacle is that the cost of WGS is comparatively high. Although the reagent cost of WGS is less than USD 1000 per case, for trio sequencing, the cost would be triplicated and was higher than routine tests. In fact, the cost would be higher to permit rapid TAT prenatally. Studies further evaluating the cost-effectiveness of WGS are warranted since the cost is falling rapidly.

In conclusion, with a rapid TAT, good diagnostic yield, and less DNA required, WGS could be an alternative test in lieu of two separate analyses as it has an equivalent diagnostic yield to that of CMA plus WES and provides comprehensive detection of various genomic variants in fetuses with structural or growth anomalies. However, more prospective studies with larger cohorts and further evaluation are warranted to demonstrate the value of WGS in prenatal diagnosis.

## Figures and Tables

**Table 1:** Summary of numerical disorder and pathogenic or likely pathogenic copy number variants (CNVs) detected by CMA and WGS.

Case ID	Clinical Indication	Serum Screening	NIPS	CMA	WGS	Added Information by WGS
2	Cystic hygroma; severe tetralogy of fallot	N	N	arr(21) × 3	seq(21) × 3	NA
40	ventricular septal defect; pulmonary atresia	N	N	arr(21) × 3	seq(21) × 3	NA
42	Ventricular septal defect	N	N	arr(13) × 3	seq(13) × 3	NA
51	Ventricular septal defect; choledochol cyst; ventriculomegaly; echogenic crystalline lens	N	Low-risk	arr[GRCh37]6q25.3q27(158678581-170914297) × 3	seq[GRCh37]dup(6)(q25.3q27)dn chr6:g.158661896-170927571dup	A pathogenic SNV (NM_005267.4,c.593G>A,p.Arg198Gln) in *GJA8* was identified by WGS
				arr[GRCh37] 8p23.3(158048-2193914) × 1	seq[GRCh37]del(8)(p23.3)dn chr8:g.156079-2185433del	
				arr[GRCh37]8p23.2p22(2347604-14015208) × 3	seq[GRCh37]dup(8)(p23.2p22)dn chr8:g.2340301-13996893dup	
52	Congenital heart disease; clubfeet; atrial septal defects; tetralogy of fallot	N	N	arr(21) × 3	seq(21) × 3	NA
84	Holoprosencephaly; ventricular septal defect; polydactyly	N	N	arr(13) × 3	seq(13) × 3	NA
93	single umbilical artery; omphalocele; umbilical cord cyst; clubhands; facial abnormality	N	N	arr(18) × 3	seq(18) × 3	NA
102-1	Skeletal dysplasia; unilateral pleural effusion; intestinal dilatation	N	Low-risk	arr[GRCh37]13q32.1q34(95443088-115107733) × 1	seq[GRCh37]del(13)(q32.1q34) chr13:g.95440312-115169878del	Arising from paternal balanced translocation
				arr[GRCh37]15q26.3(100865196-102429040) × 3	seq[GRCh37]dup(15)(q26.3) chr15:g.100877325-102531392dup	Arising from paternal balanced translocation
102-2	Fetal hydrops; ascites; pleural effusion; hydroderma	N	Low-risk	arr[GRCh37]13q32.1q34(95443088-115107733) × 1	seq[GRCh37]del(13)(q32.1q34) chr13:g.95440312-115169878del	Arising from paternal balanced translocation
				arr[GRCh37]15q26.3(100865196-102429040) × 3	seq[GRCh37]dup(15)(q26.3) chr15:g.100877325-102531392dup	Arising from paternal balanced translocation

NIPS, non-invasive prenatal screening; CMA, chromosomal microarray; WGS, whole genome sequencing; N, no screening. NA, not applicable.

**Table 2:** Summary of pathogenic or likely pathogenic single nucleotide variants (SNVs) and small insertions or deletions (INDELs) detected by WES and WGS.

Case ID	Clinical Indication	Genes	OMIM Diseases	Variants (All Heterozygous)/Inheritance	Novel or Previously Reported (PMID)
1	Oligohydramnios; renal cyst	*PKHD1*	#263200 Polycystic kidney disease 4, with or without hepatic disease (AR)	NM_138694.3, c.7994T>C, p.Leu2665Pro (Mat)	Reported (PMID30507656;PMID28851938)
				NM_138694.3, c.5428G>T, p.Glu1810* (Pat)	Novel
3	Skeletal dysplasia; micromelia	*COL1A1*	#166200/166210/259420/166220 Osteogenesis imperfecta (AD); #114000 Caffey disease (AD)	NM_000088.3, c.4280_4283delTTGA, p.Ile1427Asnfs*98 (De novo)	Novel
10	Fetal growth restriction; cerebellar dysplasia	*SBDS*	#260400 Shwachman–Diamond syndrome (AR)	NM_016038.2, c.183_184delinsCT, p.Lys62* (Pat)	Reported (PMID15769891;PMID18478597;PMID15284109)
				NM_016038.2, c.258+2T>C (Mat)	Reported (PMID15769891;PMID18478597;PMID15284109)
12	Bilateral pleural effusion; hydroderma; anhydramnios	*MUSK*	#208150 Fetal akinesia deformation sequence 1 (AR)	NM_005592.3, c.790C>T, p.Arg264* (Mat)	Novel
				NM_005592.3, c.1003_1006delGTTT, p.Val335Phefs*24 (Pat)	Novel
15	Kidney agenesis	*HNF1B*	#137920 Renal cysts and diabetes syndrome (AD)	NM_000458.2, c.494G>A, p.Arg165His (De novo)	Reported (PMID24254850;PMID27838256;PMID22051731)
16	Rhabdomyomas; subependymal nodules	*TSC2*	#613254 Tuberous sclerosis-2 (AD)	NM_000548.3, c.4258_4261delTCAG, p.Ser1420Glyfs*55 (De novo)	Reported (PMID26252095;PMID10533067;PMID29740858)
30-1	Rhabdomyomas; intracardiac echogenic focus	*TSC2*	#613254 Tuberous sclerosis-2 (AD)	NM_000548.3, c.4762C>T, p.Gln1588* (De novo)	Reported (PMID27494029)
30-2	Rhabdomyomas; intracardiac echogenic focus	*TSC2*	#613254 Tuberous sclerosis-2 (AD)	NM_000548.3, c.4762C>T, p.Gln1588* (De novo)	Reported (PMID27494029)
51 #	Ventricular septal defect; choledochol cyst; ventriculomegaly; echogenic crystalline lens	*GJA8*	#116200 Cataract 1, multiple types (AD)	NM_005267.4, c.593G>A, p.Arg198Gln (De novo)	Reported (PMID16604058)
64	Anhydramnios; hydroderma	*RYR1*	#255320 Minicore myopathy with external ophthalmoplegia (AR)	NM_000540.2, c.6082C>T, p.Arg2028* (Pat)	Novel
				NM_000540.2, c.165+5G>A (Mat)	Novel
77	Cleft lip/palate; persistent left umbilical vein	*CHD7*	#214800 CHARGE syndrome (AD)	NM_017780.3, c.2881delG, p.Glu961Serfs*16 (De novo)	Novel
97	Holoprosencephaly; agenesis of the corpus callosum	*ZIC2*	#609637 Holoprosencephaly 5 (AD)	NM_007129.3, c.916G>T, p.Glu306* (De novo)	Novel
104	Fetal growth restriction	*IARS1*	#617093 Growth retardation, impaired intellectual development, hypotonia, and hepatopathy (AR)	NM_013417.2 c.2420C>G, p.Pro807Arg (Mat)	Novel
				NM_013417.2, c.2975A>G, p.Asn992Ser (Pat)	Novel
112	Cleft lip/palate; small stomach; ventricular septal defect	*CHD7*	#214800 CHARGE syndrome (AD)	NM_017780.3, c.7153C>T,p.Gln2385* (De novo)	Novel

Mat, maternal; Pat, paternal; AR, autosomal recessive; AD, autosomal dominant; WES, whole exome sequencing; WGS, whole genome sequencing. # WGS identified a clinically relevant pathogenic SNV in *GJA8* gene in this CMA-positive case.

**Table 3:** Impact of genetic diagnoses on pregnancy outcome.

Groups	Number of Cases	Termination of Pregnancy		Continuing Pregnancy		*p* Value ^
		Number of Cases	95% CI #	Number of Cases	95% CI #	
Diagnosed	22 (19.8%)	21 (95.5%)	77.2%–99.9%	1 (4.5%)	0.1%–22.8%	0.00000053
Undiagnosed	89 (80.2%)	34 (38.2%)	28.1%–49.1%	55 (61.8%)	50.9%–71.9%	NA
Overall	111	55 (49.6%)	39.9%–59.2%	56 (50.4%)	40.8%–60.1%	NA

# 95% confidence interval was calculated by binomial exact calculation. ^ Fisher’s exact test. NA, not applicable.

## Data Availability

The data presented in this study are available on request from the corresponding author. The data are not publicly available due to privacy of the patients.

## Supplementary Materials

The following are available online at https://www.mdpi.com/2073-4425/12/3/376/s1: Table S1: Detailed clinical information of 111 fetuses. Table S2: CNVs with size ≥100 kb detected by chromosomal microarray (CMA) and/or whole genome sequencing (WGS). Table S3: CNVs with size <100 kb detected by whole genome sequencing (WGS). Table S4: Comparison of the number of rare exome variants called by whole genome sequencing (WGS) and whole exome sequencing (WES). Table S5: Prenatal detection rates of fetus groups with each phenotypic abnormality.
